# Bone Response to Weight Loss Following Bariatric Surgery

**DOI:** 10.3389/fendo.2022.921353

**Published:** 2022-07-07

**Authors:** Chiara Mele, Marina Caputo, Alice Ferrero, Tommaso Daffara, Beatrice Cavigiolo, Daniele Spadaccini, Antonio Nardone, Flavia Prodam, Gianluca Aimaretti, Paolo Marzullo

**Affiliations:** ^1^ Department of Clinical-Surgical, Diagnostic and Pediatric Sciences, University of Pavia, Pavia, Italy; ^2^ Department of Health Sciences, University of Piemonte Orientale, Novara, Italy; ^3^ Division of Endocrinology, University Hospital “Maggiore della Carità”, Novara, Italy; ^4^ Istituti Clinici Scientifici Maugeri IRCCS, Neurorehabilitation and Spinal Unit of Pavia Institute, Pavia, and Neurorehabilitation of Montescano Institute, Montescano, PV, Italy; ^5^ Department of Translational Medicine, University of Piemonte Orientale, Novara, Italy; ^6^ Istituto Auxologico Italiano, IRCCS, Laboratory of Metabolic Research, S. Giuseppe Hospital, Piancavallo, Italy

**Keywords:** bone loss, bone turnover, bone mineral density, bariatric surgery, fracture risk, rehabilitation

## Abstract

Obesity is a global health challenge that warrants effective treatments to avoid its multiple comorbidities. Bariatric surgery, a cornerstone treatment to control bodyweight excess and relieve the health-related burdens of obesity, can promote accelerated bone loss and affect skeletal strength, particularly after malabsorptive and mixed surgical procedures, and probably after restrictive surgeries. The increase in bone resorption markers occurs early and persist for up to 12 months or longer after bariatric surgery, while bone formation markers increase but to a lesser extent, suggesting a potential uncoupling process between resorption and formation. The skeletal response to bariatric surgery, as investigated by dual-energy X-ray absorptiometry (DXA), has shown significant loss in bone mineral density (BMD) at the hip with less consistent results for the lumbar spine. Supporting DXA studies, analyses by high-resolution peripheral quantitative computed tomography (HR-pQCT) showed lower cortical density and thickness, higher cortical porosity, and lower trabecular density and number for up to 5 years after bariatric surgery. These alterations translate into an increased risk of fall injury, which contributes to increase the fracture risk in patients who have been subjected to bariatric surgery procedures. As bone deterioration continues for years following bariatric surgery, the fracture risk does not seem to be dependent on acute weight loss but, rather, is a chronic condition with an increasing impact over time. Among the post-bariatric surgery mechanisms that have been claimed to act globally on bone health, there is evidence that micro- and macro-nutrient malabsorptive factors, mechanical unloading and changes in molecules partaking in the crosstalk between adipose tissue, bone and muscle may play a determining role. Given these circumstances, it is conceivable that bone health should be adequately investigated in candidates to bariatric surgery through bone-specific work-up and dedicated postsurgical follow-up. Specific protocols of nutrients supplementation, motor activity, structured rehabilitative programs and, when needed, targeted therapeutic strategies should be deemed as an integral part of post-bariatric surgery clinical support.

## Introduction

Obesity is a disease (1) and a global health challenge, now included among the global noncommunicable disease targets identified by the World Health Organization (2). Bariatric surgery constitutes a remarkable tool against obesity and its comorbidities (3). The current epidemic proportions reached by obesity make it a cornerstone treatment to contrast obesity if resistant to standard weight-loss approaches, especially when significant weight loss results are mandatory to control obesity-related impaired health conditions. A number of recent clinical practice guidelines exist on bariatric surgery in adults with obesity (4–6) and the evolution of surgical trends in the past 10 years shows similarities and disparities in the number and types of surgical and endoluminal interventions (7). As for trends of standard bariatric surgery, sleeve gastrectomy (SG) shows a continuous upward trend, while Roux-en-Y gastric bypass (RYGB) and laparoscopic adjustable gastric band (AGB) have trended downward (5). In parallel, an increasing number of gastroenterologists are performing bariatric endoscopic procedures that include placement of intragastric balloons, plications and suturing of the stomach, and insertion of a duodenal-jejunal bypass liner, among other emerging procedures (8).

To ensure long-term postoperative success, patients must be prepared to adopt comprehensive lifestyle changes, yet a number of endogenous and exogenous factors are known to influence bariatric surgery outcomes, as summarized in **Table 1** (5, 9–25). It is known that postsurgical weight loss is associated with extended health benefits in terms of arterial hypertension, diabetes mellitus, cardiopulmonary problems, dyslipidemia, susceptibility to neoplasms, osteoarticular disabilities, gastroesophageal reflux disease, psychosocial wellbeing, quality of life, as well as decreased odds of cardiovascular fatalities, strokes and all-cause-mortality (26–29). Nevertheless, there is growing interest on the potential impact of bariatric surgery on bone health, as changes have accumulated to suggest the postsurgical development of accelerated bone loss and skeletal fragility (30). After gastric bypass, an increase in bone resorption markers occurs as early as 10 days postoperatively (31), then marker levels peak by 6 to 12 months (31, 32) and remain elevated thereafter (33). Biochemical markers of bone formation increase but to a lesser extent, suggesting a potential “uncoupling” of resorption from formation (34–37). Several post-bariatric surgery mechanisms have been claimed to occur and act globally on bone health, including mechanical unloading due to bodyweight loss, adipocyte-derived and gastro-enteric hormone changes, and malabsorptive factors (30, 34, 37, 38). Concerns exist on the key role of bariatric surgery on micronutrient intake, diminished calcium and vitamin D absorption leading to secondary hyperparathyroidism, and restricted energy delivery (30, 37). Secondary hyperparathyroidism, which often occurs before bariatric surgery due to the highly prevalent vitamin D deficiency in obesity, progressively increases its prevalence following bariatric surgery, going from 21% at baseline to 35.4% at 1 year and 63.3% at 5 years after surgery (39). The mechanism resides in calcium malabsorption which, in association with vitamin D deficiency, promotes secondary hyperparathyroidism leading to bone resorption. There are even claims that, in the very long term, post-bariatric surgery hypocalcemia may overstimulate parathyroid gland and lead to the anectodical development of a parathyroid adenoma (40). Further, intestinal adaptation mechanisms, local effect of the Wnt/β-catenin signaling pathway, changes in carrier proteins, and the effect of adipokines are other calcium-related mechanisms potentially involved in bone loss after bariatric surgery (40, 41).

**Table 1:** Baseline and peri-operative factors associated with higher and more durable total weight loss after bariatric surgery.

Factors	Outcomes
Age	Younger patients tend to experience greater results than elderlies
Gender	Higher absolute weight loss occurs in men, greater BMI loss in women
Presurgical bodyweight	Higher preoperative BMI (particularly super-obesity) is associated with less weight loss
Surgical approach	Effectiveness on total weight loss varies as follows: BPD > RYGB > SG > AGB
Motivations and expectations	Physiological, emotional, cognitive, and interpersonal/environmental factors can strengthen bariatric surgery outcomes
Eating behaviors	Disordered eating is associated with poorer weight loss and greater weight regain in the long term
Adherence to dietary guidelines	Presurgery nutritional evaluation, dietary adherence and postsurgery nutritional follow-up are associated with successful postsurgical weight loss.
Gastroenteric environment	Gut hormones, bile acids and gut microbiota predict responses to successful weight loss
Sarcopenia	No interaction is suggested between obesity sarcopenia and postsurgical skeletal muscle loss as compared to nonsarcopenic persons
Muscle mass maintenance and propensity to physical exercise	Physical activity after bariatric surgery is associated with enhanced weight loss outcomes

AGB, laparoscopic adjustable gastric banding; BMI, body mass index; BPD, bilio-pancreatic diversion; RYGB, Roux-en-Y gastric bypass; SG, sleeve gastrectomy.

While the skeletal response to the effects of bariatric surgery has been collectively confirmed in systematic reviews and meta-analyses, discrepancies still exist in terms of affected bone regions and timing of dynamic bone changes (30, 42, 43). As such, dual-energy X-ray absorptiometry (DXA) studies have shown significant BMD loss at the hip with less consistent results for the lumbar spine (44, 45). Despite weight stabilization and maintenance of metabolic parameters, bone loss and deterioration in bone strength continued years following bariatric surgery, supporting the hypothesis that fracture risk does not appear to be dependent on weight loss, and rather it increases with observation time (46). Moreover, an increased risk of fall injury has been reported after bariatric surgery, which contributes to increase the fracture risk in patients undergoing bariatric surgery procedures (47, 48). Supporting DXA studies, analyses by high-resolution peripheral quantitative computed tomography (HR-pQCT) also showed lower cortical density and thickness, higher cortical porosity, and lower trabecular density and number for up to 5 years after gastric bypass (49). Given these circumstances, it is conceivable that bone health should be accounted for before deciding on the type of bariatric surgery. Little is known about the role of lifestyle intervention, rehabilitation and pharmacological treatments to prevent or treat post-bariatric surgery bone loss and fracture outcome.

The aim of the present review is to update the current data regarding the mechanisms and determinants of bone damage after bariatric surgery and the strategies of prevention and treatment.

## Crosstalk Between Obesity and Bone Health

**Figure 1:**
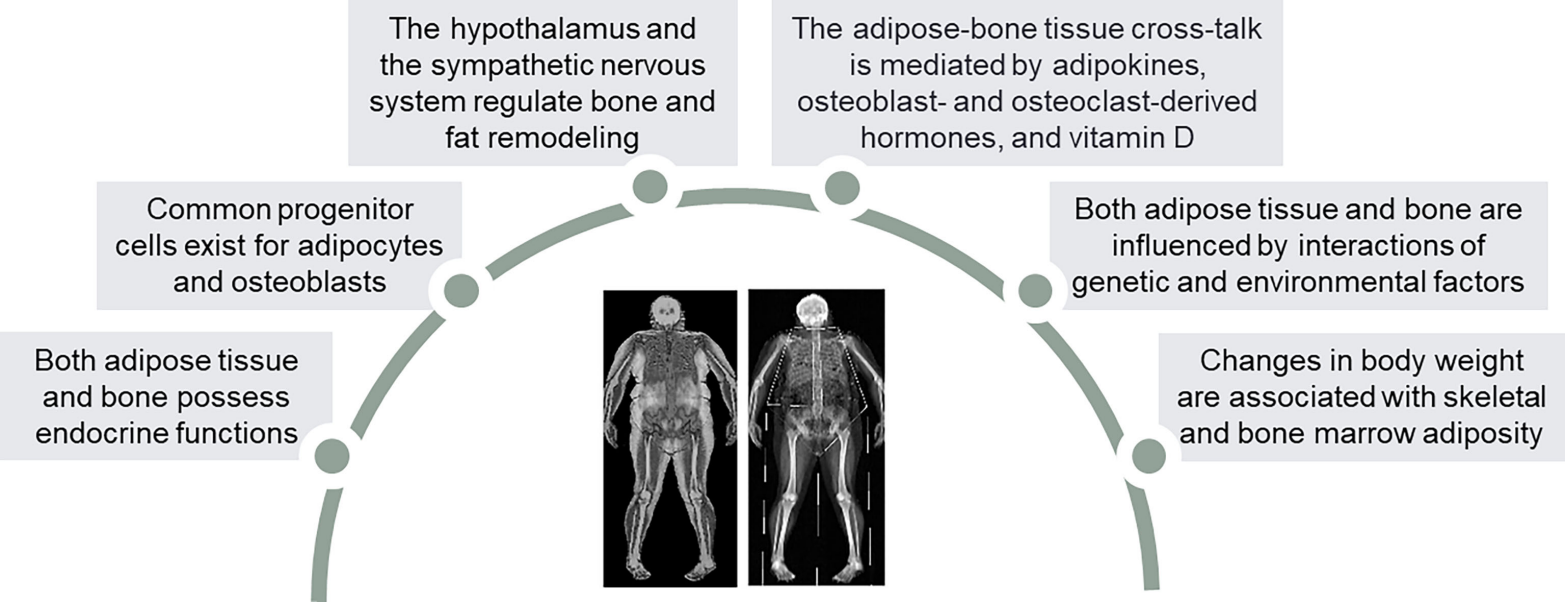
Similarities and homologies between adipose tissue and bone.

The crosstalk between adipose tissue and bone is regulated by a large number of interacting factors (**Figure 1**). A common stromal cell origin of osteoblasts and adipocytes has been proposed as a possible link between adipose tissue and bone (50). Despite being acknowledged as beneficial to bone mineral density (BMD) due to the mechanical loading effect of weight excess (51), obesity is consistently emerging as a potential detrimental factor for bone health, particularly appendicular bones. Studies highlighted the impact of fat overload on bone strength (52–54) and cortical rearrangement through insulin resistance (55). Osteoporosis is associated with obesity in one out of three women (56), and nearly one out of four postmenopausal women with fractures can present with obesity, particularly in the case of ankle and femur fractures (57). Further, femoral neck BMD is reduced and risk of non-vertebral fragility fractures increased in obese postmenopausal women compared to lean counterparts (58). Collectively, potential mechanisms claimed to explain the interaction between obesity, menopause and bone metabolism (59) include: 1) visceral fat accumulation (60) and sarcopenia (61) compromise the mechanical loading effect (62); 2) lower vitamin D levels and secondary hyperparathyroidism can favor osteoporosis (63); 3) obesity affects endocrine somatotroph, adrenal and thyroid signals active on the bone (64–66); 4) increased risk of type 2 diabetes mellitus (T2DM) can impair femoral neck strength (67, 68).

It is noteworthy that several molecules can serve as signaling triggers between adipose and bone tissue. These include several products and determinants, i.e. adipocytokines released by the adipose tissue (AT) and its macrophage-rich stromal fraction, osteoblast- and osteoclast-derived proteins, as well as several vitamins (69–75). Leptin and other adipokines secreted by the adipose tissue can modulate bone cells through major inhibition of bone remodeling, whereas molecules activating the peroxisome proliferator-activated receptor-γ can drive mesenchymal stem cell differentiation from osteoblastic towards adipocyte lineage (76). Obesity-associated leptin resistance has been linked to decreased bone mass, as seen in case of hypoleptinemia due to extreme leanness (77). Leptin actions involve inhibition of osteoblastic bone formation through its binding to a specific receptor located in the hypothalamus (69, 70). A study conducted by Ducy et al. showed that leptin receptor expression is associated with noradrenalin release and activation of β2 adrenergic receptor in osteoblasts, thus reducing their activity (70). In mice, leptin also inhibits endocrine function of osteoblasts by sympathetic enhancement of the Esp gene expression, thereby decreasing osteocalcin bioactivity and leading to hyperinsulinemia and glucose intolerance (78).

Importantly, insulin partakes in the feedback loop between pancreas and osteoblasts (79), enhances bone remodeling and promotes the decarboxylation of osteocalcin. Osteoblast-derived osteocalcin circulates both as carboxylated (cOC) and undercarboxylated (ucOC) isoforms (69). ucOC possesses extra-skeletal effects, as it stimulates insulin expression and secretion, β-cells proliferation and adiponectin expression in adipocytes, thus resulting in improved glucose tolerance (69, 80). In 1998, Rosato et al. (81) reported that osteocalcin levels were lower in patients with T2DM than healthy subjects, while others observed lower serum osteocalcin levels in patients with poor metabolic control as compared to those with adequate metabolic control and to healthy subjects (82). In rodents and humans, ucOC has also been found to reduce fat mass accumulation through an increase in adiponectin production (83–88). In osteocalcin-deficient homozygous mice (Ocn -/-), Lee et al. (71) documented higher blood glucose and lower insulin levels than in wild-type or heterozygous mice. More, Ocn -/- mice showed an increase in fat mass, adipocyte number and serum triglyceride levels than wild-type mates. In children and adolescents with obesity, serum osteocalcin is reported to be inversely associated with markers of metabolic health and meta-inflammation, i.e. HOMA-IR, HbA1c, triglycerides, C-reactive protein, and fibrinogen, as well as with measures of adiposity, i.e. body mass index (BMI), body fat and waist circumference (87).

Recent studies emphasized a potential role for the osteocyte-secreted product of the SOST gene, sclerostin, in the relationship linking adiposity, T2DM and bone (89, 90). Biologically, sclerostin antagonizes the Wnt/β catenin signaling pathway, which regulates positively osteoprogenitor cell and osteoblast activity (91–93), and plays a role in adipocyte differentiation and metabolic homeostasis (94). Sclerostin predicts bone loss in relation to age, gender and menopause (95), prolonged immobilization (96), and postmenopausal hip fracture risk (97). Even if no difference appears to exist between obese and controls (98), serum sclerostin was found to be negatively associated with insulin sensitivity in obese but not lean subjects, suggesting a potential role for the Wnt/β-catenin pathway in regulating insulin sensitivity in obesity (99). Moreover, sclerostin is increased in states of unloading and possibly mediates changes in bone metabolism associated with weight loss and exercise (100). A negative association relates sclerostin to skeletal muscle mass after adjusting for multiple confounders (101). Finally, in pre- and postmenopausal women with obesity sclerostin positively predicted lumbar spine BMD. Although this relationship seems to conflict with the intrinsic osteopenic effects of sclerostin, this finding suggests a potential role of this hormone in the protective effects elicited by obesity on lumbar spine at menopause (102). Further studies are required to clarify this issue.

Another relevant link between bone, adipose tissue and glucose metabolism is vitamin D. It has been demonstrated that 25-hydroxyvitamin D (25(OH)D) concentrations are positively associated with adiponectin (103) and negatively associated with indices of insulin resistance (103, 104), BMI, and leptin (105). Debatedly, the adipose tissue (AT) is capable of storing vitamin D and, in case of AT excess, it is deemed as responsible of leading to a reduction in its circulating levels (106, 107). Moreover, there is suggestion that vitamin D deficiency promotes greater adiposity by elevating PTH release, which has been shown to increase intracellular calcium accumulation in adipocytes, thereby enhancing lipogenesis (108). After cholecalciferol administration, a change in multimeric adiponectin is also seen (109). Adiponectin is regulated by osteocalcin and has insulin-sensitizing effects (110), with a well-known negative correlation with parameters of the metabolic syndrome (111–114).

In summary, the crosstalk between bone metabolism and adipose tissue involves multiple factors, which could exert different regulatory mechanisms that affect the skeletal health. However, these mechanisms still need to be clarified.

## Bone Turnover Markers After Bariatric Surgery

Bariatric surgery is characterized by rapid and dramatic changes in body composition and nutritional factors that are paralleled by changes in bone turnover markers (37, 115). Bone turnover can be inspected and monitored through circulating markers that include serum C-terminal cross-linked telopeptide of type I collagen (CTX), procollagen type I N-terminal propeptide (PINP), bone-specific alkaline phosphatase (BALP), and osteocalcin.

**Figure 2:**
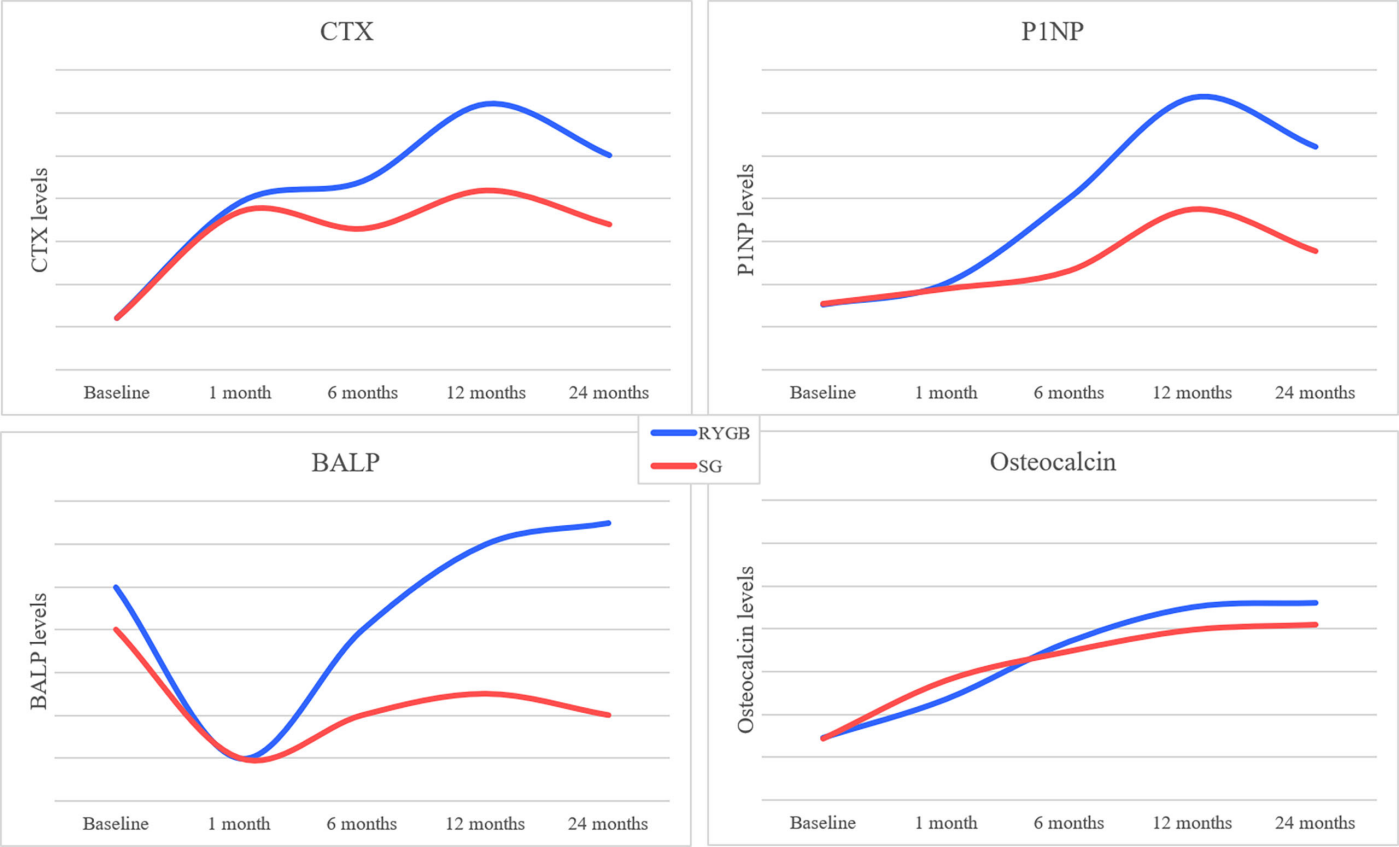
Visual graph of changes over time of bone turnover markers levels after bariatric surgery procedures (RYGB and SG). Data extracted from the references (116, 118,120).

These factors dramatically increase from the first 3-10 days, peak after 6-24 months, and remain elevated until 7 years following bariatric surgery (116–118). Both SG and RYGB promote increases in bone turnover markers, with the latter eliciting the strongest effects and leading to an increase in CTX by 50%-300% (37, 49, 119). Comparative analyses between RYBP and SG showed a significantly higher increase in CTX, P1NP, TRAcP5b with the former, and a greater increase in total OC and uOC with the latter, suggesting a predominating bone resorption over bone formation markers during RYGB (36). A randomized triple-blind trial showed an approximately 100% higher increase in P1NP and CTX-1 levels after RYGB than SG at 1-year post surgery (45). Likewise, studies after biliopancreatic diversion (BPD) showed that CTX increased significantly at 3 days (+ 66%), 3 months (+ 219%), and 12 months (+ 295%), while OC decreased at 3 days (- 19%) then increased at 3 months (+ 69%) and 12 months (+ 164%), suggesting an earlier and greater increase in bone resorption over bone formation markers with BPD (120). Although both CTX and P1NP start to decline after 12-24 months since surgery, they do not tend to return to presurgical levels (116, 121). Oppositely, BALP is a less varying bone turnover marker (10-25%) which predominantly changes during the first year after surgery and more after RYGB than SG (49, 118, 119). **Figure 2** summarizes visually the changes occurring over time in bone turnover markers after bariatric surgery. Interestingly, the magnitude of variation in bone turnover markers is reportedly similar between diabetic and non-diabetic cohorts undergoing bariatric surgery, yet patients T2DM patients carry per se a higher risk of osteoporosis (122, 123). Because alterations in CTX and P1NP levels have also been reported in adolescents after RYGB (124) and SG (125), uncertainties remain on the final effect of bariatric surgery on bone mass peak and subsequent adult risk of osteoporosis.

## Bone Mineral Density After Bariatric Surgery

A negative impact of bariatric surgery on bone mineral density (BMD) has been described since the 1980s. Hey et al. described the case of a 38-year old woman who underwent a jejunoileal bypass for severe obesity. Despite vitamin D supplementation, five years after the surgical procedure, the patient develop a fragility fracture of the distal forearm, which healed only after intestinal reanastomosis. The authors hypothesized that the surgery-related malabsorption could lead to alterations in bone metabolism with an increased fracture risk (126). Several studies and meta-analyses on the relationship between bariatric surgery and bone loss have clearly demonstrated that significant declines in BMD occur already within the first year after bariatric surgery (127). Following any type of bariatric surgery, the BMD at the femoral neck has been found significantly lower as compared to controls, with a mean difference (MD) of -0.05 g/cm2 (95% CI -0.07 to -0.02) (43). Oppositely, no difference in BMD was found at column between the two groups.

Growing evidence suggested that the negative effect of bariatric surgery on BMD is strictly dependent on the type of the surgical procedure. A systematic review and meta-analysis by Rodriguez-Carmona et al. demonstrated a significant decrease of -0.03 g/cm2 (95% CI -0.06 to 0.00) in total BMD in patients undergoing mixed restrictive-malabsorptive surgical procedures, but not in those undergoing restrictive surgery (128). In particular, BMD was reduced by -0.07 g/cm2 (95% CI -0.11 to -0.03) at the lumbar spine, -0.12 g/cm2 (95% CI -0.15 to -0.10) at the hip and -0.03 g/cm2 (95% CI -0.04 to -0.02) at the forearm.

BMD changes following bariatric procedures differ depending on skeletal sites and time passed since the operation. At the hip, bone loss following RYGB reaches -3 to -5% after 6 months (129–132) and -6 to -11% after 9-12 months (34, 35, 46, 133–135). Similar results were reported for bone loss at the femoral neck, with a decrease in BMD of -1 to -5% after 6 months and -2 to -9% after 9-12 months, mostly in patients undergoing malabsorptive procedures (34, 35, 38, 49, 130–138). A decline in BMD at the hip and femoral neck has also been observed after restrictive procedures, equivalent to -2 to -8% after 6-24 months (130, 131). At the lumbar spine, some authors did not observe significant variations in BMD at 6-12 month after RYGB (34, 129, 135), whereas others showed a significant reduction in BMD equivalent to -2 to -6% at 6 months and -3 to -7% at 9-12 months (33, 133, 134, 138). Nogues et al. observed a mild difference in BMD loss at the lumbar spine between SG and RYGB (-4.6% vs -6.3%, respectively) (139), while others reported a significant reduction of BMD at this level only after restrictive procedures (130, 140). Further, Maghrabi and co-workers conducted a randomized controlled trial on patients with T2DM to evaluate BMD after 2 years since RYGB and SG, as compared to intensive medical treatment (141). At the hip, BMD loss was significantly higher in patients undergoing SG (-9.2%) and RYGB (-9.5%) than intensive medical therapy group (-0.3%), whereas at lumbar spine a significant decrease in BMD was only observed in the SG group (-2.3%), without changes in RYGB (0.4%) and intensive medical therapy groups (0.8%). A subsequent randomized controlled trial in patients with obesity and T2DM demonstrated that subjects undergoing RYGB had a higher decrease in BMD at the femoral neck (mean between-group difference -2.8%, 95% CI -4.7 to -0.8), total hip (mean between-group difference -3.0%, 95% CI -5.0 to -0.9) and lumbar spine (mean between-group difference -4.2%, 95% CI -6.4 to -2.1) than patients undergoing SG (45).

Overall, the decrease in BMD mainly occurs in the first years after bariatric surgery during the period of rapid weight loss, but it continues even after reaching a stable weight, suggesting that the impact of bariatric surgery on BMD is not completely explained by weight loss (142). Losses appear to be heavier after malabsorptive surgery. Several mechanisms have been hypothesized to explain the negative effects of bariatric surgery on bone metabolism, including mechanical unloading, malabsorption of macro and micronutrients, alterations in gut-derived hormones and microbiota, and changes in body composition (30, 37), which have been extensively described in the following paragraphs.

## Bone Micro-Architecture After Bariatric Surgery

As seen in many observational studies and clinical trials investigating bone loss associated with bariatric surgery, attention is primarily focused on areal BMD (aBMD) evaluated by using dual-energy X-rays absorptiometry (DXA). However, in the setting of a marked weight loss, DXA results could be influenced by changes in the composition of the bone surrounding tissues (37, 143). In addition, DXA is unable to assess bone microarchitecture, as it cannot discriminate trabecular from cortical bone compartments (37). With the aim of overcoming such limitations, more recent studies have focused on HR-pQCT to evaluate post-bariatric volumetric BMD (vBMD) and bone microarchitecture (38, 46, 119, 135, 144–146). A pioneer study by Stein and co-workers (38) assessed HR-pQCT at the distal radius and tibia in 22 women who underwent RYGB (n=14) and restrictive procedures (n=8). Compared to baseline, after 12 months from surgery trabecular parameters remained stable in both sites, while cortical bone deterioration was observed as exemplified by reductions at the tibia in cortical density (-1.7%), cortical thickness (-2.1%) and cortical area (-2.7%). These changes in cortical compartment were noticed to be more pronounced after RYGB and were independently predicted by the increase in PTH levels, thus suggesting a preferential endocortical bone resorption (38).

In a prospective cohort study on 30 obese adults and 20 non-surgical controls, Yu et al. evaluated the rate of bone loss and microarchitectural alterations occurring in 24 months after RYGB (144). Their results showed that total vBMD progressively decreased after surgery both at the radius and tibia, with a 9% decrease of bone strength as compared to the control group. At the radius, the decrease in bone strength was associated with a greater reduction in trabecular vBMD together with an increase in trabecular heterogeneity, while similar reductions in cortical and trabecular vBMD were seen at the tibia. In addition, the authors observed that the impairment in bone microarchitecture, density and strength observed after the first 12 months was maintained or even worsened after 24 months following surgery, despite a bodyweight plateau reached after 6 months. This temporal connection highlights the potential complex origin of bone loss associated with bariatric surgery.

**Figure 3:**
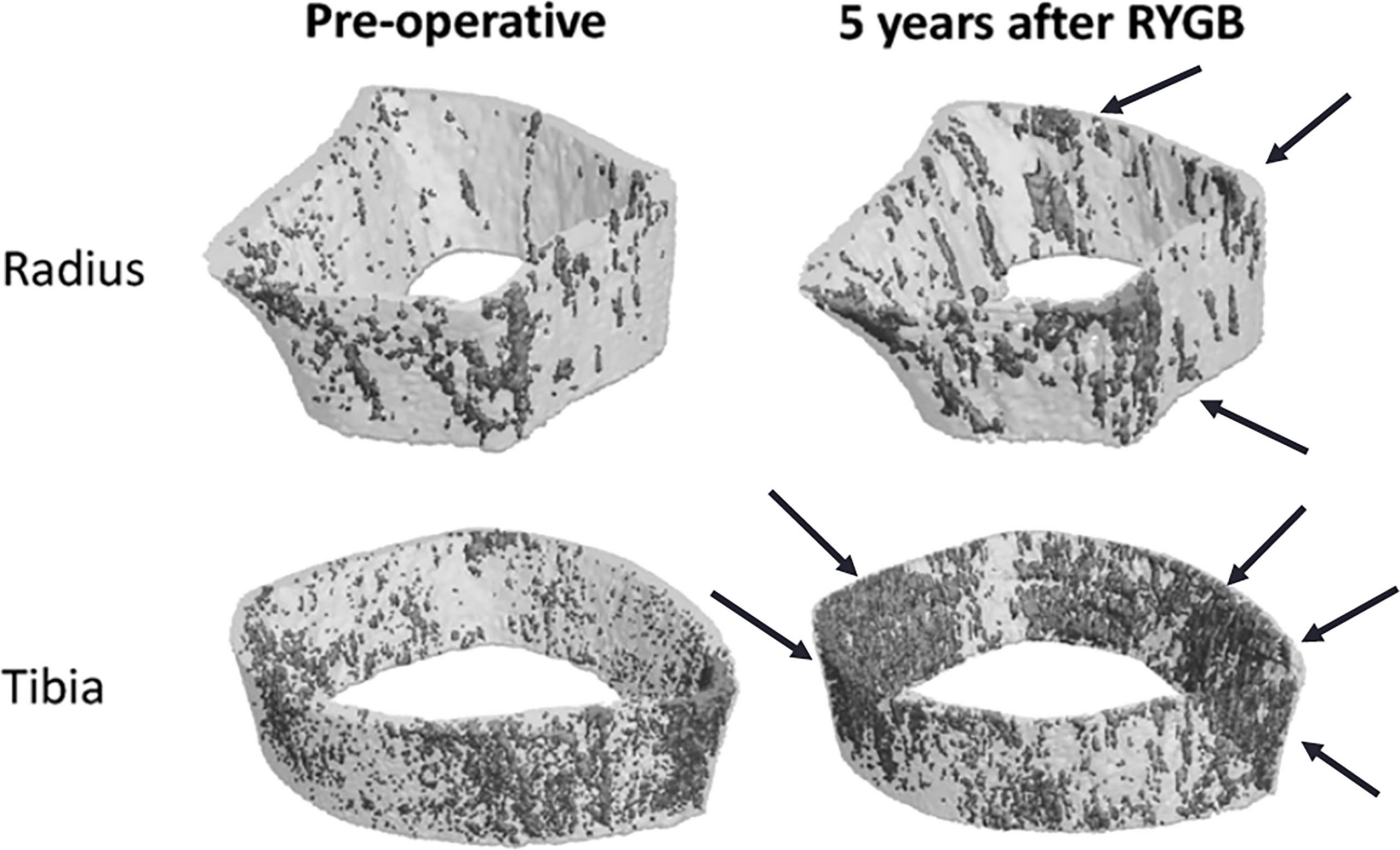
Prospective 5-year observational study of cortical porosity at the distal radius and tibia after RYGB in 21 adults with severe obesity. Declines in cortical and trabecular microarchitecture led to decreases in estimated failure load of -20% and -13% at the radius and tibia (46).

Subsequent studies (46, 119, 135, 145), underscored an increased porosity in the bone cortical compartment associated, in some instances, with a significant decrease in cortical and trabecular vBMD both at the radius and tibia (46, 49, 119, 135, 145) (**Figure 3**). The effects of bariatric surgery on bone microarchitecture and vBMD are early and occur within the first months, with a progressive deterioration over the following years (46, 119, 135, 145). It has been also hypothesized that the changes in the cortical compartment of weight bearing and non-weight bearing sites could be modulated by estrogen concentrations in the bone microenvironment (46, 135).

A recent comparative study on vBMD and bone microarchitecture 10 years after RYGB and adjustable gastric banding (AGB) in comparison to age, sex and BMI-matched non-surgical controls (146) documented that total vBMD at the tibia and radius were 17% and 19% lower than in controls, respectively. Alterations were prominent in trabecular microarchitecture and consisted of lower trabecular number and thinner trabeculae. Moreover, in RYGB group, trabecular bone was less axially aligned at both radius and tibia with a decrease in plate bone volume fraction and density as compared to matched controls. No significant differences were found in terms of bone morphology and microarchitecture between the AGB group and controls (146).

In summary, all the evidences confirmed that the decrease in vBMD after bariatric surgery is strongly related to a significant deterioration in bone microarchitecture and strength, thus predisposing to greater bone fragility.

## Fracture Risk After Bariatric Surgery

**Figure 4:**
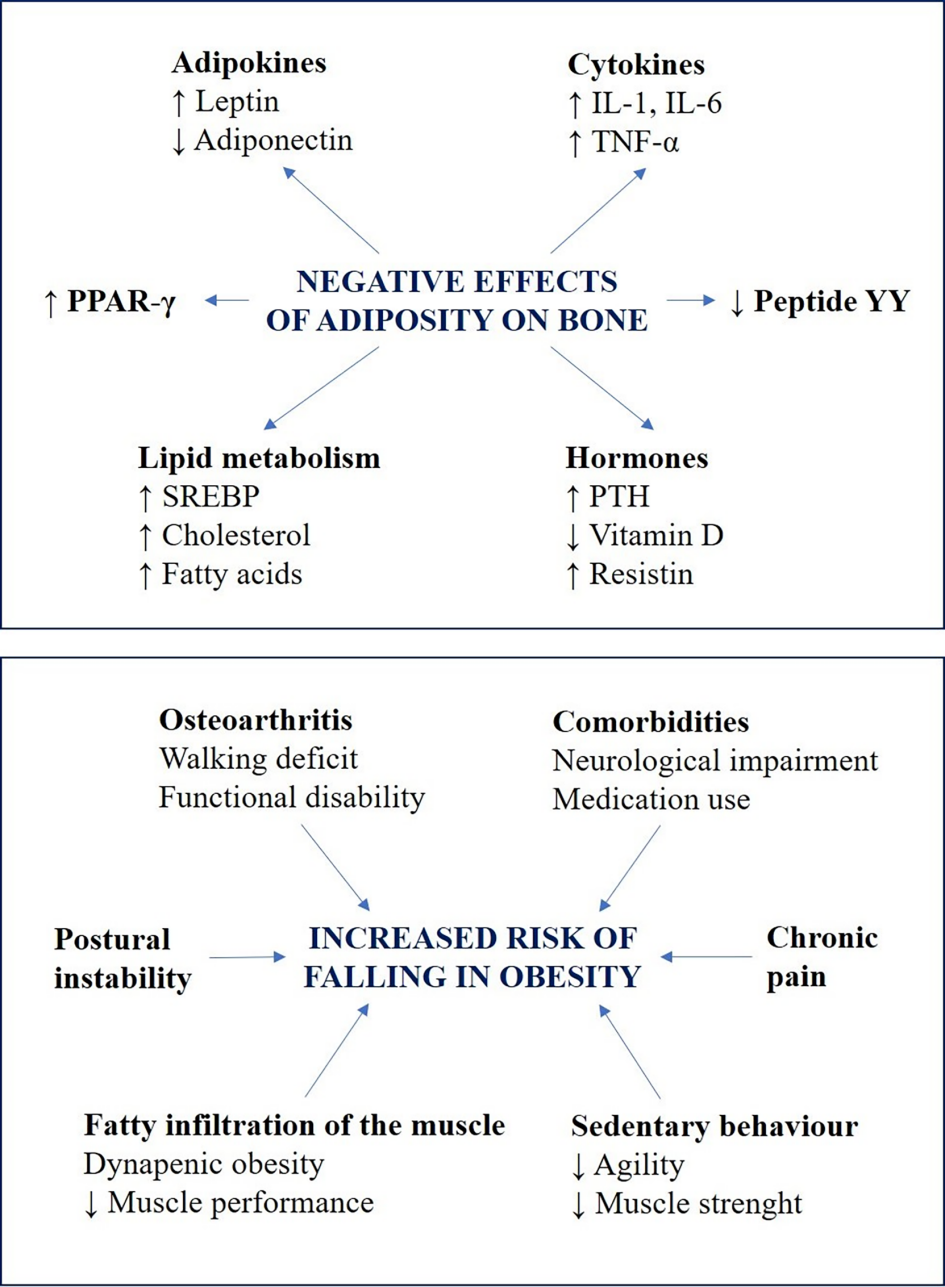
Summary of the two hypothesized mechanisms to explain the susceptibility of obese patients to bone fractures. The negative effects of adiposity on bone fragility are reported in the upper box: obesity is associated with alterations in adipokines and cytokines levels, deregulation of peptides and hormones related to bone metabolism, and dyslipidaemia (112, 146). All these factors contribute to alter bone resorption and formation, by acting directly on osteoclast and osteoblast or indirectly through different molecular pathways. The factors that influence the risk of falling in obesity are reported in the lower box: the mechanistic links between falls and obesity include chronic health conditions, medication use and sedentary behaviour, which lead to a reduction in muscle strength and agility (147). Moreover, biomechanical alterations including poor muscle quality, impaired postural control and osteoarthritis, may reduce postural stability and muscle performances, thus inducing walking deficit and functional disability (148–150).

Despite the large body of evidence suggesting that obesity is associated with an increase in BMD, both relating to higher estrogens levels in the adipose tissue and due to the mechanical effect of weight increment (115), this increase in BMD does not reflect a functional improvement of bone microarchitecture, and several studies have demonstrated a higher risk of fracture in obese patients (147). This “obesity paradox” (148) has been attributed to mechanisms involving an increase in bone fragility caused by adiposity and higher risk of falls (115, 149–153) (**Figure 4**).

Opposed to what would be expected, weight loss is not associated with a decrease in bone fractures risk. In fact, some authors have demonstrated that weight loss, both unintentional and intentional, leads to a decrease in BMD at the proximal humerus and the hip (154–156), with a consequent increase of fracture risk in these sites (157–159). Ensrud and co-workers demonstrated that women achieving intentional and non-intentional weight loss had 1.8 times the risk of subsequent hip fracture (95% CI 1.43–2.24) than those with stable or increasing weight (154). Moreover, a decline of 35% in hip BMD for every 5 kg lost has been observed. The consequences of bariatric surgery are strictly connected with the effect of weight loss, and many studies suggested its potential negative effects on bone metabolism (160). A summary of the results of observational studies and interventional trials is reported in **Table 2** (48, 141, 161–169).

**Table 2:** Summary of the observational and interventional studies on the fracture risk after bariatric surgery.

References	Study design	Participants	Type of surgery	Follow-up (years) Mean (SD) or Median (IQR)	Fracture risk for bariatric surgeryrisk ratio (95% CI)
Lalmohamed A 2012 (158)	Retrospective cohort study	Bariatric surgery: 2079 Control group: 10442	AGB: 1249 RYGB: 613 Other: 217	Bariatric surgery: 2.2 (2.1) Control group: 2.3 (2.2)	Adjusted RR (bariatric vs control group): - any fracture: 0.89 (0.60-1.33) - fragility fracture: 0.67 (0.34-1.32) - non-fragility fracture: 0.90 (0.56-1.45)
Nakamura KM 2014 (159)	Retrospective cohort study	Bariatric surgery: 258 No control group	RYGB: 243 VBG: 13 BPD: 1 PBD: 1	Bariatric surgery: 8.9 (4.8) Control group:/	SIR (bariatric vs control group): - any fracture: 2.3 (1.8-2.8) - osteoportic sites: 2.0 (1.3-3.0) - non-osteoporotic sites: 2.4 (1.8-3.0)
Douglas IJ 2015 (160)	Retrospective cohort study	Bariatric surgery: 3882 Control group: 3882	GB: 1829 RYGB: 1421 SG: 613 GS: 6 SP: 5 DS: <5 VBG: <5	Bariatric surgery: 3.4 (2.3) Control group: 3.4 (2.4)	HR for any fracture (bariatric vs control group): 1.28 (0.81-2.02)
Lu CW 2015 (161)	Observational cohort study	Bariatric surgery: 2064 Control group: 5027	Malabsorptive: 289 Restrictive: 1775	Bariatric surgery: 4.8 (2.3) Control group: 4.9 (2.1)	Adjusted HR for any fracture (bariatric vs control group): - all procedures: 1.21 (1.01-1.44) - malabsorptive procedures: 1.47 (1.01-2.15) - restrictive procedures: 1.17 (0.97-1.41)
Maghrabi AH 2015 (138)	Randomized control trial	Bariatric surgery: 37 Control group: 17	SG: 19 RYGB: 18	12 and 24 months	RR for peripheral fractures (bariatric vs control group): 2.12 (0.44-10.16)
Rousseau C 2016 (45)	Case-control study	Bariatric surgery: 12676 Control group: - Obese: 38028 - Non-obese: 126760	AGB: 3887 SG: 2554 BPD: 1986 RYGB: 873	4.4 (range <1-13)	Adjusted RR for any fracture: - Bariatric vs non-obese group: 1.44 (1.29-1.59) - Bariatric vs obese group: 1.38 (1.23-1.55)
Fashandi AZ 2018 (162)	Retrospective cohort study	Bariatric surgery: 3439 Control group: 3380	RYGB: 2729 AGB: 385 SG: 268 Other: 57	From 3 to 22 years	OR for any fracture (bariatric vs control group): - all procedures: 2.36 (1.72-2.23) - RYGB (vs SG): 2.17 (1.04-4.52)
Javanainen M 2018 (163)	Retrospective cohort study	Bariatric surgery: 395 Control group: 199	RYGB: 253 SG: 142	12 and 24 months	HR for any fracture (bariatric vs control group): 5.49 (1.76-17.15)
Yu EW 2019 (164)	Retrospective cohort study	Bariatric surgery: 42345	RYGB: 29624 AGB: 12721	RYGB: 3.3 (2.2) AGB: 3.9 (2.1)	Adjusted HR for non-vertebral fractures (RYGB vs AGB): 1.73 (1.45-2.08)
Ahlin S 2020 (165)	Nonrandomized controlled intervention study	Bariatric surgery: 2007 Control group: 2040	VBG: 1365 GB: 376 RYGB: 266	From 6 months to 20 years	Adjusted HR for any fracture: - VBG vs controls: 1.20 (1.00-1.43) - GB vs controls: 1.30 (0.97-1.74) - RYGB vs controls: 2.58 (2.02-3.31) Adjusted HR for osteoporotic fractures: - VBG vs controls: 1.15 (0.87-1.51) - GB vs controls: 1.85 (1.27-2.70) - RYGB vs controls: 3.60 (2.56-5.05)
Khalid SI 2020 (166)	Retrospective cohort study	Bariatric surgery: 32742 Control group: 16371	RYGB: 16371 SG: 16371	3 years	OR for any fractures: - RYGB vs controls: 0.95 (0.84-1.07) - SG vs controls: 0.53 (0.46-0.62) - RYGB vs SG: 1.79 (1.55-2.06)
Paccou J 2020 (167)	Retrospective cohort study	Bariatric surgery: 40992 Control group: 40992	SG: 18635 RYGB: 14532 AGB: 5178 VBG: 2647	Bariatric surgery: 6.19 years Control group: 5.26 years	Adjusted HR for major osteoporotic fractures: - Bariatric surgery vs controls: 1.22 (1.08-1.39) - SG vs controls: 0.95 (0.79-1.14) - RYGB vs controls: 1.70 (1.46-1.98) - AGB vs controls: 0.95 (0.72-1.25) - VBG vs controls: 0.95 (0.68-1.31)
Alsaed OS 2021 (168)	Case-controlled study	Bariatric surgery: 403 Control group: 806	SG: 334 RYGB: 69	8.6 years (mean)	OR for any fracture: 2.71 (1.69-4.36)
Chin WL 2021 (169)	Retrospective cohort study	Bariatric surgery: 1322 Non-surgical group: 1322 General population: 4359	Not specified	87.55 months (median)	Adjusted HR for any fracture: - Bariatric surgery vs non-surgical group: 0.77 (0.54-1.11) - Bariatric surgery vs general population: 2.21 (1.57-3.11) Adjusted HR for non-traffic accident-related fractures: - Bariatric surgery vs non-surgical group: 0.54 (0.34-0.87) - Bariatric surgery vs general population: 1.69 (1.08-2.66)

AGB, adjustable gastric banding; RYGB, roux-en-Y gastric bypass; VBG, vertical-banded gastroplasty; BPD, biliopancreatic diversion; PBD, pancreatobiliary diversion; SIR, standardized incidence ratios; GB, gastric band; SG, sleeve gastrectomy; GS, gastric stapling; SP, stomach partition; HR, hazard ratio; OR, odds ratio; SG, sleeve gastrectomy.

One of the first studies designed to evaluate the increased fracture risk in patients undergoing bariatric surgery was published in 2014 by Nakamura and co-workers (162). The authors observed that the relative risk (RR) for any fracture was increased by 2.3-fold as compared to non-surgical controls and that the standardized incidence ratios (SIRs) for a first fracture in osteoporotic sites, including the spine, hip, wrist, or humerus, was nearly doubled (SIR, 1.9; 95% CI, 1.1-2.9). Many subsequent retrospective and interventional studies confirmed the strong association between bariatric surgery and bone fractures risk, also in long-term follow-up (47, 48, 164–166, 168, 170, 171). A recent meta-analysis of 10 observational studies was conducted to compare the fracture risk between 116,205 subjects who underwent bariatric surgery and 134,637 non-surgical patients (121). The results showed that the risk of any fractures was significantly increase by 20% in the group of bariatric surgery than the control counterpart. Despite the low rating on the risk of bias assessment scales, the analysis on three interventional trials shows a trend toward an increase in the fracture risk in patients who underwent bariatric surgery (RR 1.16, CI 95% 1.00-1.33).

Alternatively, Lalmohamed at al. failed to observe a significant increase in the fracture risk in patients undergoing bariatric surgery as compared to control group (adjusted relative risk 0.89, 95% CI 0.60-1.33). However, the authors found a trend towards an increased fracture risk after 3 to 5 years following surgery and in patients with a greater weight loss (161). A lack of association between bariatric surgery and an increased fracture risk was also observed in three subsequent retrospective cohort studies (163, 169, 172). The results of a randomized controlled trial aiming at investigating the 2-year outcomes of bariatric surgery vs intensive medical therapy, have reported a lower total and hip BMD in bariatric surgery but the number of bone fractures did not differ between groups (141). It is important to note that these studies predominantly included patients who underwent restrictive surgery with few cases of malabsorptive procedures (161, 163). Some suggested that the degree of damage to bone microarchitecture varies according to the type of surgery (48, 162). Paccou and co-workers, in a population-based cohort study including 81,948 patients (40,992 in the bariatric surgery group, and 40,992 matched controls), observed a 70% increased risk of fragility fractures only for RYGB within 10-year after surgery (170), while no association between the risk of fragility fractures and SG, AGB and vertical banded gastroplasty (VBG) was seen. An average 1.4-fold higher fracture risk was also observed in a Bayesian network metanalysis, with differences emerging across the various surgical procedures (173), as subjects receiving mixed restrictive/malabsorptive procedures tended to suffer from an increased risk of fracture as compared with those undergoing restrictive procedures (RR 1.54, 95% CI 0.96-2.46). A meta-analysis by Chaves et al. confirmed that malabsorptive procedures elicited a high fracture risk as compared to controls (RR 1.53, CI 95% 1.13-2.07), and RYGB group had a higher risk as compared to SG group (RR 1.77, CI 95% 1.48-2.12) (118). It has been suggested that the higher fracture risk related to malabsorptive or combined procedures could be attributable to neurohormonal changes and malabsorption (37).

## Potential Mechanisms Associated With Bone Loss After Bariatric Surgery

A number of mechanisms have been hypothetically linked to postsurgical bone loss, which may involve nutrient absorption deficits, mechanical unloading, alterations in bone marrow adipose tissue (BMAT), as well as changes in adipokines and gut-derived hormones (**Table 3**) (30, 92, 174–195).

**Table 3:** Summary of the mechanisms hypothesized to explain the negative effects of bariatric surgery on bone metabolism.

Factors	Mechanisms	Changes after bariatric surgery	Expected effect on bone
Mechanical loading	The skeletal adaptation to mechanical strain and loading is fundamental to preserve bone mass and microarchitecture. Weight loss reduces mechanical loading on bone structure, thus favoring bone loss (171).	↑ Mechanical unloading	Upregulation of bone turnover/BMD loss
Nutritional factors			
*Vitamin D/calcium*	A high prevalence of hypovitaminosis D have been documented in obese patients (172). Vitamin D deficiency is worsened by bariatric surgery and calcium absorption is impaired, particularly after malabsorptive procedures (30).	↓ Vitamin D levels ↓ Calcium levels ↑ PTH	Upregulation of bone turnover/BMD loss
*Amino-acids*	The early post-surgery phase after malabsorptive procedures is characterized by protein depletion (173).	↓ Muscle mass ↑ amino-acids levels	BMD loss
Neuroendocrine and gut-derived hormones			
*Peptide YY (PYY)*	PYY is produced and secreted by the enteroendocrine L-cells of the colon and ileum to counteract caloric intake (174). Serum concentrations of this hormone increase are directly associated with a higher bone turnover (175).	↑ PYY levels (in contrast with conventional weight loss)	BMD loss
*Glucose-dependent insulinotropic polypeptide (GIP)*	The incretin hormone GIP is produces and secreted from duodenum and jejunum (176). Animal studies have shown that GIP increase in bone formation. To date, studies with the aim of investigating the post-surgical changes of this hormone in correlation with BMD variations are lacking (30).	↓ GIP levels	BMD loss
*Glucagon-like peptide type 1 (GLP-1)*	The incretin hormone GLP-1 is produced and secreted by the enteroendocrine L-cells located in the distal ileum and colon. Only few studies correlate the post-bariatric increase in GLP-1 levels with bone metabolism. A recent interventional study has suggested that GLP-1 variations after bariatric surgery do not significantly affect bone metabolism (177).	↑ GLP-1 levels (in contrast with conventional weight loss)	No significant role in bone turnover and BMD loss
*Ghrelin*	Ghrelin is a 28-amino acid peptide mainly released from the oxyntic cells of the stomach mucosa in response to fasting. While *in vitro* ghrelin promotes osteoblast differentiation and inhibits osteoclastogenesis, in (178), in humans there is no association between BMD and ghrelin levels (179).	↓ Ghrelin levels (in contrast with conventional weight loss)	No significant role in bone turnover and BMD loss
*Amylin*	Amylin is a pancreatic hormone with pleiotropic effects in different organs. It stimulates osteoblasts activity and inhibits bone reabsorption (180).	↓ Amylin secretion	Upregulation of bone turnover/BMD loss
*Insulin*	Insulin is secreted by pancreatic beta cells and represents a potential regulator of bone metabolism, considering that insulin receptors are expressed on osteoblasts (181). While *in vitro* studies have shown that insulin promotes osteoblast proliferation and differentiation (182), insulin signaling in human osteoblasts stimulates bone reabsorption by reducing osteoprotegerin levels (183). However, associative studies have shown that insulin levels are directly associated with bone density (181).	↓ Insulin levels	Upregulation of bone turnover/BMD loss
Adipokines and other hormones			
*Adiponectin*	Adiponectin is secreted by adipose tissue and is negatively associated with fat mass. Observational studies have reported that adiponectin levels are negatively correlated to BMD (184, 185).	↑ Adiponectin	BMD loss
*Leptin*	Leptin is secreted by adipose tissue and its circulating levels are positively associated with fat mass. This peptide regulates energy expenditure and plays a pivotal role in bone metabolism, by increasing bone formation and reducing bone resorption (181).	↓ Leptin levels	Upregulation of bone turnover
*Visfatin*	Visfatin is a multifaced adipokine whose serum levels are increased in obese subjects and associated with insulin resistance (186). Observational studies did not find any association between visfatin concentrations and BMD (187, 188).	↓, ↑ or ↔ Visfatin levels	Unclear role
*Sclerostin*	Sclerostin is the osteocyte-product of the SOST gene and represents a major inhibitor of the osteogenic Wnt signaling pathway (89).	↑ Sclerostin levels	BMD loss
*Estrogen*	Obesity is characterized by hyperestrogenism and weight loss induces a significant reduction in total and free estradiol. Estrogens exert a fundamental role in promoting osteoblastic activity and in regulating bone turnover (189).	↓ Estrogen levels	Upregulation of bone turnover/BMD loss
Body composition	Bariatric surgery is characterized by a decrease of both fat mass and muscle mass (190, 191). The relationship between the loss of muscle mass and the impairment of bone health is widley known.	↓ Muscle mass	Alterations in bone microarchitecture/BMD loss
Bone marrow adiposity (BMA)	Contrary to what expected, BMA is increased in weight loss and is related to a lower BMD and vertebral fractures (192).	↑ BMA	BMD loss

↑, increased; ↓, reduced; ↔, unchanged.

In 1985, Parfitt et al. described bone histomorphometric changes in patients undergoing intestinal bypass and collected evidence from literature of 2.5-25% rate of osteomalacia after shunt operations, commenting that osteomalacia after intestinal bypass surgery has similar clinical, biochemical and histologic features as in other causes of net intestinal malabsorption (196). Bariatric surgery impairs the ability of the digestive tract to secrete hydrochloric acid required for digestion and absorption of nutrients which are required for bone formation and healthy bone remodeling, such as trace elements, essential minerals, water-soluble and fat-soluble vitamins. Many of these disorders are inadequately replaced after surgery, especially if extended gastric bypass surgery, duodenal switch or biliopancreatic diversion are involved (197). Among these, calcium is absorbed passively as well as actively in the small intestine, and a study using a dual stable calcium isotope method (198) demonstrated a significant reduction in calcium absorption following RYGB despite adequate vitamin D and calcium intake. With concern to vitamin D, even solely restrictive procedures like SG can lead to postoperative vitamin D deficiency in as much as 39% of patients despite daily multivitamin supplementation (199), while malabsorptive surgeries pose a higher risk for nutrient deficiencies (200). In a retrospective study, a 73% incidence of vitamin D deficiency was observed following BPD (201).

Weight loss following bariatric surgery instigates a condition of mechanical unloading, that causes a net loss of bone mass by reducing osteoblast function and bone formation. The mechanism likely involves the aforementioned sclerostin and its negative regulation of Wnt/β-catenin signaling pathway, which is important for osteoblast differentiation and function (202). Maintenance of bone strength and density is indeed dependent on adequate muscle mass and function, which reflects intake of high-quality protein (203). Bariatric patients ingest less than 60–120 g of protein recommended daily (197, 204). The combination of postsurgical malnutrition, negative skeletal muscle protein balance, and rapid weight loss initiates a net loss of fat-free mass especially during the first postoperative year, which can last up to 36 months or even up to 9 years post-surgery, and is correlated with loss of handgrip strength (205). Post-operative decrease in fat-free mass and fat mass does not differ between RYGBP and LSG (206). While the biomechanical interaction between nutrition, muscle and bone after bariatric surgery is of key relevance, also important is the biochemical communication existing between muscle and bone which involves secreted factors that act bidirectionally with autocrine/paracrine effects locally as well as through the endocrine system (207). Together, these mechanisms and cross-talks potentially represent the theoretical basis to explain the bone-directed benefit of protein supplementation and mechanical loading through exercise after bariatric surgery. In addition to the previous, a role for bone marrow adipose tissue (BMAT) has also suggested in this scenario. Identified over 100 years ago, BMAT represents up to 70% of bone marrow volume in humans and is a metabolically active, insulin-sensitive and molecularly distinct fat depot that may play a role in whole body energy metabolism (208). Several factors are known to impact bone marrow fat such as obesity, T2DM, estrogen deficiency, caloric restriction, aging, chronic kidney disease, radiotherapy and glucocorticoids. In obese individuals, BMAT fraction is negatively associated with aBMD and the change described for BMAT fraction following RYGB is positively associated with changes in BMI and total body fat (209). RYGB decreases both BMAT and vBMD, yet their changes are unrelated (210).

**Figure 5:**
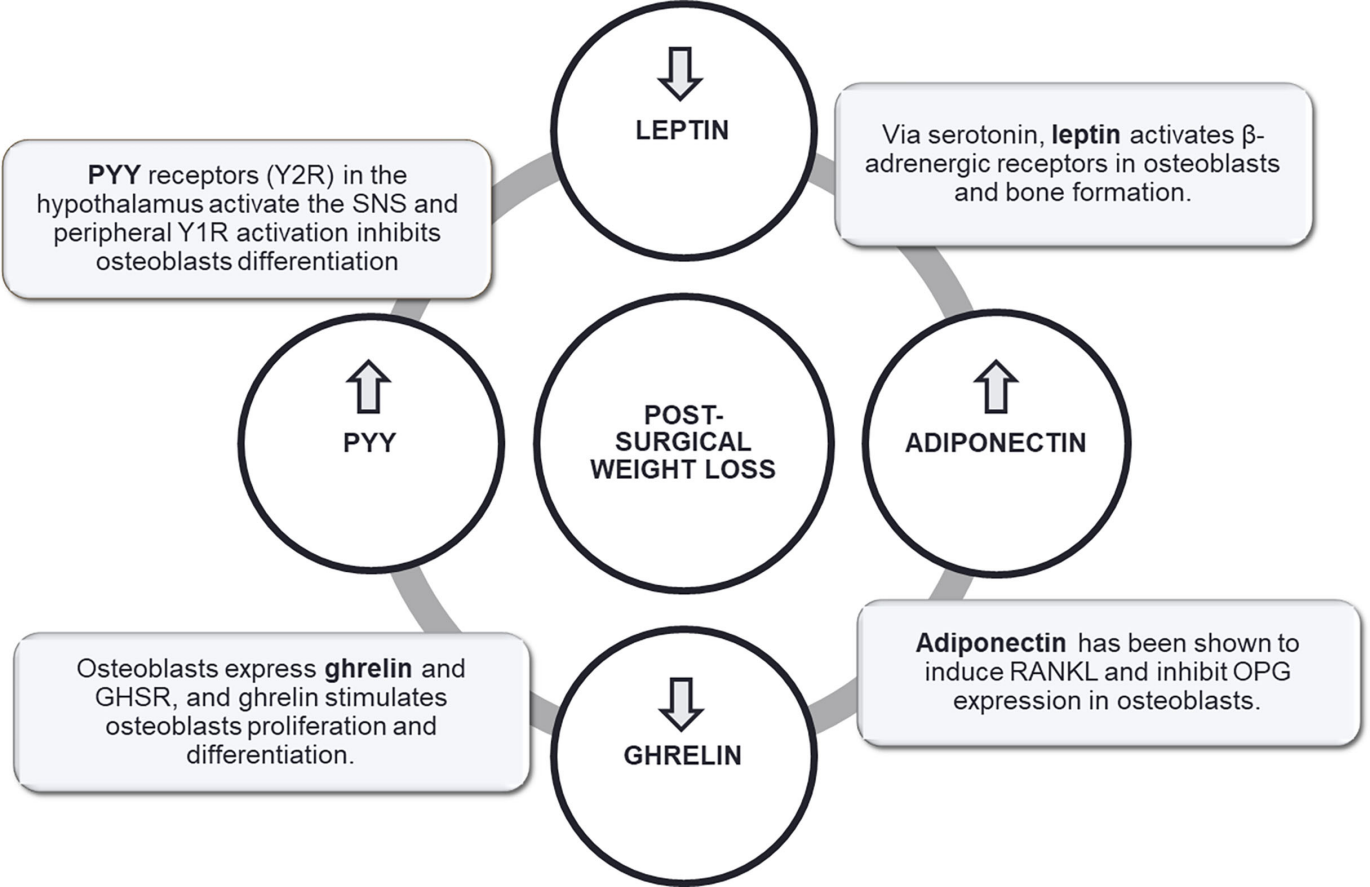
Putative mechanisms linking post-bariatric surgery weight loss to changes in bone cells.

Finally, a role for adipokines and gut hormones through their central and peripheral receptors has been described (211–216), with evidence indeed coming mainly from animal and *in vitro* studies (**Figure 5**).

In humans, bariatric surgery modulates a change in gut hormones, the magnitude of which is dependent of the surgical technique and has a predictive value on postsurgical weight loss and metabolic improvements by mechanisms other than restriction and malabsorption (24). In rodents, GLP-1 administration elicited an anabolic effect on bone, while GLP-2 was associated with reduction in bone resorption markers (217, 218). *In vitro*, it was further observed that ghrelin administration suppressed osteoclastogenesis and stimulated proliferation and differentiation of osteoblasts (219), while a study in bariatric surgery patients reported an association between ghrelin reduction and BMD loss after RYGB and SG (220). Also, PYY has been positively associated with bone resorption (221), whereas a positive correlation linking PYY to CTX and P1NP levels was observed in patients following RYGB and AGB. In a 12-month study on patients subjected to RYGB, SG or greater curvature plication, postsurgical bone mineral content at the lumbar spine was inversely correlated both with fasting ghrelin and GLP-1, while BMD was positively correlated with post-surgical fasting glucagon and insulin at the femoral neck and inversely with GLP-1 at the lumbar spine.

## Supplementation and Antiresorptive Treatments

### Supplementation Treatment

In the last two decades, increasing evidence has showed that post-bariatric surgery patients need a specific protocol of supplementation in order to prevent bone loss and the risk of osteoporosis in the following years.

Calcium balance and vitamin D levels are considered as the main components for maintaining bone mass after bariatric surgery. Nevertheless, a high proportion of subjects do not reach an adequate intake, especially after the surgery. It is estimated that, 4 years following a malabsorptive bariatric procedure, calcium deficiency develops in 25–48% of patients, and vitamin D deficiency in 50–63% of cases. For this reason, before administration of antiresorptive agents, normal vitamin D and calcium levels should be guaranteed even by means of aggressive supplementation, especially when hypocalcaemia and hyperparathyroidism are present (127, 222). In general, GB is associated with higher prevalence of hyperparathyroidism and hypocalcaemia compared to SG and therefore the recommended doses are typically higher. On the other hand, patients subjected to BPD/duodenal switch (DS) show the highest needs in terms of supplementation (222, 223).

As far as the recommended doses are concerned, guidelines established a minimal daily intake of 1,200-1,500 mg/day for SG and RYGB, and 1,800-2,400 mg/day for BPD with or without DS of elemental calcium in the diet or as supplement (5, 222). Calcium citrate is generally recommended over calcium carbonate and should be given in divided doses to enhance absorption.

For vitamin D, a recent meta-analysis reported that at least 800 UI/die can be sufficient to maintain a level of vitamin D sufficiency (224). Due to the high heterogeneity of patients, therapies should start from 1000 UI/die and go up to 2,000-6,000 IU daily, depending on the malabsorption level. A target vitamin D level of 30 ng/ml is desirable. In the case of severe vitamin D malabsorption, an initial oral dose of vitamin D equivalent to 50,000 IU should be administered 1 to 3 times weekly (5, 204, 222). In case of secondary hyperparathyroidism, the Endocrine Society Clinical Practice Guidelines suggested that a weekly 100,000 IU of parenteral ergocalciferol could be useful, until the target Vitamin D level ≥ 30 ng/ml is achieved, resorting to calcitriol if bone loss or elevated PTH persisted (5).

Magnesium, has also shown to be slightly increased in blood after gastric bypass, even if it requires HCl from secreting parietal cells to be solubilized and its absorption is compromised due to the reduction of fatty acids which are bound with. However, the supplementation of at least 100 mg/day of magnesium is highly recommended since its increase in blood, like calcium, could be associated to a higher bone resorption (225–227).

### Antiresorptive *Treatments*

In spite of the negative bone effects of bariatric surgery, the optimal medical management for these patients has not been elucidated yet (5). In fact, use of antiresorptive therapy (i.e., bisphosphonates and denosumab) is potentially burdened by a high risk of adverse events in this particular population (228).

The major risks for oral bisphosphonates are reflux and anastomotic ulceration (229). On the other hand, the administration of intravenous bisphosphonate and denosumab may be complicated by severe hypocalcemia and tetany in patients without adequate calcium or vitamin D levels.

According to clinical practice guidelines of American Association of Clinical Endocrinologists, the Obesity Society, and the American Society for Metabolic and Bariatric Surgery for the peri-operative nutritional, metabolic and nonsurgical support of bariatric surgery (224), bisphosphonates may be a considered in bariatric surgery patients affected by osteoporosis after appropriate assessment and treatment for calcium and vitamin D insufficiency. Moreover, if oral malabsorption is suspected or potential anastomotic ulceration risk is evaluated, intravenously bisphosphonates should be preferred. Recommended dosages of orally and intravenous administered bisphosphonates in bariatric surgery patients with osteoporosis are summarized in **Table 4**. In spite of this, no clinical trial data regarding the use of bisphosphonates in post-bariatric patients are available to date.

**Table 4:** Recommended dosages of orally and intravenously bisphosphonates in bariatric surgery patients affected by osteoporosis.

Type of bisphosphonates	Route of administration	Dose	Frequency
Alendronate	os	70 mg	week
Risedronate	os	35 mg	week
	os	150 mg	month
Ibandronate	os	150 mg	month
	iv	3 mg	3 months
Zoledronate	iv	5 mg	year

The risk related to malabsorption is the failure in reaching the optimal blood level and the to obtain the therapeutic effect. If malabsorption is suspected, a safe choice should be risedronate, as a pharmacokinetic study in non-bariatric surgery patients demonstrated that it was absorbed along the small bowel independently of the site of administration (stomach, duodenum or terminal ileum), and the range of absorption is not affected by the rate of administration (aqueous solution or iv infusion) (230). A trial on risedronate in sleeve gastrectomy patients is ongoing (NCT03411902), and study design only has been published (231).

Considering the risk of gastric ulceration, studies in non-bariatric patients demonstrated that bisphosphonates differ in their potential effect in damaging the gastroesophageal mucosa. In fact, in postmenopausal women received 5 mg risedronate or 10 mg alendronate daily for 2 weeks, risedronate was associated with fewer endoscopically detected gastric ulcers than alendronate (232), probably related to the structural differences in the nitrogen-containing group.

Thus, intravenously bisphosphonates have been proposed for bone management in postoperative bariatric patients. The safety considerations for zoledronate 5 mg once a year and ibandronate 3 mg every three months includes the onset of flu-like syndrome (low-grade fever, muscle and joint pain) and renal adverse events, particularly in high-risk patients (i.e., dehydration, concomitant nephrotoxic medications, myeloma kidney) (233). Recently, a pilot study explored the safety and efficacy of zoledronic acid in preoperative post-menopausal women who were planning RYGB: a single dose of zoledronate appeared to transiently (2 weeks) reduce bone turnover markers, but at 24 weeks after surgery an increase in CTX versus baseline was observed, although the rise was less than that observed in the controls, even without differences in total hip BMD (234). Moreover, a trial to determine the efficacy of zoledronic acid in preventing bone loss associated with sleeve gastrectomy is ongoing (NCT04279392).

A major concern in the administration of intravenously bisphosphonates is the occurrence of hypocalcemia, in particular after gastric bypass; thus, adequate vitamin D level should be ensured because a bypassed small bowel may not be able to absorb calcium enough to compensate the effects of bisphosphonate binding to bone matrix (235).

In this framework, data on denosumab are even more scanty. A randomized placebo-controlled trial to establish the role of denosumab to prevent bone loss after RYGB or sleeve gastrectomy is ongoing (NCT04087096). The same observations of iv bisphosphonates regarding the risk of hypocalcemia should be considered for denosumab treatment (236).

## Physical Exercise and Rehabilitation

As previously mentioned, bariatric surgery induces a decline in muscle mass, which is responsible for the 10-28% of total body weight loss (237, 238). A prospective cohort study on 184 patients who underwent SG showed that the prevalence of sarcopenia increases from 8% up to 32% within one year after surgical procedure (239). Muscle waist and sarcopenia were found to be independently associated with several adverse outcomes, including functional decline, a higher rate of falls as well as a higher risk of hospitalization and mortality (OR 3.596, 95% CI 2.96-4.37) (240). Therefore, during follow-up of patients after bariatric surgery, the assessment of BMD, muscle mass and strength, physical activity and fitness should be considered (241). With the aim at improving these parameters, individual interventions of physical activity and rehabilitation have been proven to be effective in preserving muscle mass and endurance capacity, in reducing the risk of bone fractures and in improving quality of life (242–245).

In this context, several studies have demonstrated that physical activity during weight loss is able to prevent the reduction of BMD (100, 246, 247). In 2016, Muschitz et al. conducted an interventional study in 220 patients after RYGB and SG procedures with the aim of assessing the differences in serum markers of bone turnover and BMD between an intervention group (supplementation of vitamin D, calcium, protein, and physical exercise) and a non-intervention group after 2 years from surgical procedure (248). The physical activity intervention consisted of an aerobic and strength exercise program including Nordic walking for 45 minutes for at least 3 times a week and strength training for 30 minutes for at least 2 times a week, for two years. The results showed that the supplementation combined with physical exercise exert a positive effect on long-term outcome in bone protection after bariatric surgery, by modulating serum levels of sclerostin, CTX, DKK-1 and PTH, and counteracting the loss of the spine, hip and total body BMD. One year later, Campanha-Versiani and co-workers evaluated the role of physical exercise after RYGB in influencing BMD and bone turnover markers in a group of patients undergoing a regular and supervised exercise program compared to a control group (249). In this study, physical exercise combined weight-bearing and aerobic exercises two times a week for 36 weeks. One year following RYGB, the intervention group showed a lower decrease in total BMD and at the lumbar spine and hip than the control group, without significant differences in terms of serum concentrations of bone remodeling markers.

Recently, Diniz-Sousa et al. conducted a systematic review and meta-analysis to extensively evaluate the effect of physical exercise and training on BMD at clinically relevant skeletal sites during the first year after bariatric surgery (250). Meta-analysis showed that the decrease in BMD after bariatric surgery can be attenuated from 0.7 to 3.7 percentage points with an exercise training intervention. In particular, exercise training induced a positive effect on BMD at femoral neck [standardized mean difference (SMD)=0.63 (95% CI 0.19-1.06)], total hip [SMD=0.37 (95% CI 0.02-0.71)], lumbar spine [SMD=0.41 (95% CI 0.19-0.62)], and 1/3 radius [SMD=0.58 (95% CI 0.19, 0.97)] as compared to standard medical interventions.

Overall, the evidences suggest that physical exercise after bariatric surgery is fundamental, as it prevents bone loss and muscle depletion during the drastic weight reduction period (142, 205, 251). Exercise programs that include high-impact loading, resistance and strength training as well as aerobic exercises seem to be effective in counteracting the negative effects of bariatric surgery on BMD and bone microarchitecture (142, 252, 253).

Being bariatric surgery patients exposed to multiple systemic risks and particularly for cardiovascular diseases and musculoskeletal impairment, a tailored rehabilitation program through a multidisciplinary approach is key to optimize post-bariatric surgery management (254). The multidisciplinary approach integrates different clinical specialties including endocrinology, clinical nutrition, psychiatry, rehabilitation medicine, as well as health professionals such as nursing, physiotherapy and occupational therapy (255). A post-bariatric surgery rehabilitation project should comprise a number of goals: 1) preventing surgical-related complications, 2) enhancing physical function through adapted physical activity, 3) addressing bariatric-related disabilities as well as socio-environmental and psychological barriers, 4) promoting education on nutritional management, and 5) providing primary or secondary prevention for cardiovascular diseases (254). The rehabilitation program should thus combine musculoskeletal reconditioning, functional mobility, balance training, muscle strengthening, aerobic exercises or physical endurance, activity of daily living (ADL) training, nutritional and psychological support, weight management, and monitoring of clinical aspects (254).

## Conclusions

Bone changes after bariatric surgery may have effects that go well beyond the acute phase of weight loss. Bariatric surgery, especially malabsorptive procedures, increases bone turnover, decreases bone mass, and enhances the fracture risk. These consequences advocate the need to adequately study, monitor and support skeletal health and micronutrient supply in bariatric surgery patients to avoid short- and long-term damage in bone density. There is collective evidence that bone density estimation by DXA can be improved by HR-pQCT to better classify patients at risk of osteoporosis. To compensate for nutritional and mechanical deficits after surgery, replenishment with calcium citrate and high-dose vitamin D plus scheduled exercise programs are mandatory. Noticeably, absorption problems and potential ulceration of anastomosis should be considered before prescribing oral bisphosphonates. In patients who fail to achieve BMD improvements post-surgically, intravenous bisphosphonates and/or denosumab should be considered, with calcium and vitamin D being critical to avoid hypocalcemia. Yet, RCTs are needed to determine whether anti-osteoporotic therapy is effective and safe for preventing high-turnover bone loss and treating osteoporosis in this population. Finally, it is of utmost importance to consider bone health before deciding the type of bariatric surgery a patient with obesity should be subjected to.

## Author Contributions

Conceptualization and methodology, CM, MC, and PM. Original draft preparation, CM, MC, AF, TD, BC, DS, and FP. Review and editing, CM, GA, and PM. Supervision, GA, AN, and PM. All authors contributed to the article and approved the submitted version.

## Funding

Research founded by the Italian Ministry of Health (18C101_2011).

## Conflict of Interest

The authors declare that the research was conducted in the absence of any commercial or financial relationships that could be construed as a potential conflict of interest.

## Publisher’s Note

All claims expressed in this article are solely those of the authors and do not necessarily represent those of their affiliated organizations, or those of the publisher, the editors and the reviewers. Any product that may be evaluated in this article, or claim that may be made by its manufacturer, is not guaranteed or endorsed by the publisher.
